# The impact of vaccination upon dental clinic avoidance and the cessation of individual protection measures

**DOI:** 10.3389/fpubh.2022.864783

**Published:** 2022-09-21

**Authors:** Maria Jose González-Olmo, Rafael Gómez de Diego, Bendición Delgado-Ramos, Martin Romero-Maroto, María Carrillo-Diaz

**Affiliations:** ^1^Department of Dentistry, Rey Juan Carlos University, Madrid, Spain; ^2^Department of Dentistry, Granada University, Campus Universitario de Cartuja, Granada, Spain

**Keywords:** coronavirus infections, COVID-19, infectious disease transmission, professional-to-patient, perceived vulnerability to disease, disease avoidance, dental care

## Abstract

The aim of this study was to analyze the evolution of germ aversion, to perceived infectability and to the fear of COVID-19 from the beginning of the pandemic until the arrival of the vaccines. A repeated measures design was used with three time points during the pandemic. The survey consisted of: Scale of perceived vulnerability to disease; Scale of fear of COVID-19; They were asked if they were vaccinated and if that vaccination is complete. They were asked if they would avoid the dental clinic through fear of COVID-19; and if they have reduced preventive practice in response to COVID-19. A T0-T1 increase in perceived infectability and germ aversion was reported. However, fear of COVID-19 decreased at T1-T2. The vaccinated experienced a greater reduction than the unvaccinated and a greater relaxation of their preventive practice. The frequency of dental avoidance decreased in the vaccinated group from T1 to T2 by 68.3% while in the non-vaccinated this reduction was only 4.9%; *X*^2^ = 18.58 (*p* < 0.01). In summary, vaccination has had an impact in the reduction of perceived infectability and in reducing fear of COVID-19. Nevertheless, germ aversion has remained stable and independent of vaccination. Empirical support is found for the affirmation that vaccination can reduce certain preventive behavior and dental avoidance.

## Introduction

The SARS-CoV-2 virus infection (COVID-19) (1) first appeared in China at the end of 2019 and in a few months became a global threat. It was proclaimed a pandemic by the World Health Organization in March 2020 (2). In an effort to reduce the transmission of the virus and the probability of contracting the illness, policies of mitigation control were introduced (3): obligatory mask wearing, disinfection of hands, cleaning of frequently touched surfaces, social distancing, mobility restrictions and time limitations on non-essential activity (4–6). This imposed a drastic change in the daily behavior of citizens who in general followed the guidelines adequately. However, strict obedience to preventive measures has been influenced by the fear of COVID-19 and an aversion to germs in general (7, 8).

A priori, germ aversion could prove to have positive results by facilitating the identification of possible sources of pathogens and by encouraging avoidance behavior, which could lead to a subsequent reduction in the likelihood of infection (9). Nevertheless, germ aversion may become a real germ phobia and into a highly incapacitating disorder called mysophobia (8).

As well as preventive hygienic measures, the implementation of lockdown in homes and the over-information of the mass media had an impact on physical health and in matters of a psycho-social nature and upon the economy (6, 10). There has been a dramatic decrease in medical consultations, caused by the fear of infection of COVID-19. This lowered rate of attendance may result in aggravated episodes of serious pathology at home, which can have irreversible consequences for patients' health as much at a systemic level as dental (11–13). On the other hand, there are some areas in which telemedicine was essential and the pandemic has accelerated the process, easing the population the access to the sanitary system.

Until now these protective measures have been able to slow the progression of the virus, although the most hopeful strategy for successfully achieving the reduction of levels of mortality and morbilidad continue being vaccination and the development of effective, safe and accessible medicines. By May 2, 2021 a total of 17, 309, 914 vaccination doses (14) have been administered in Spain, of which the percentage in Madrid where the study has been centered has reached 31.2% of the population with one dose and 11.9% with the complete two injections (15). It is apparent that the required herd immunity (estimating the threshold of collective immunity to oscillate between 50 and 67% (16, 17) is still far from being achieved based on current numbers. The acceleration of the vaccination rate against SARS-CoV-2 is encouraging and the population has glimpsed an end to the pandemic. It remains unknown however if the reduction in risk perception will affect the continuation of preventive behavior, which could suppose a change in dental attendance.

As well, this study has as its objective the analysis of the evolution of germ aversion, to perceived infectability and to the fear of COVID-19 from the beginning of the pandemic until the arrival of the vaccines. The impact that vaccines play in modifying the fear of COVID is also evaluated as is any change of attitude with regard to preventive conduct in response to COVID-19 and to dental avoidance.

## Materials and methods

### Design type

A repeated measures design was used with three time points: before lockdown (T0), after completion of total lockdown (T1) and when the vaccination process begins in certain risk groups, some essential workers, and the population over 65 years of age (T2).

A self-completed questionnaire was administered to a convenience sample of residents in a district of Madrid (a representative area of the community in socioeconomic terms). In T0, which had not yet declared a state of alarm in Spain or the lockdown (March 1–March 8, 2020) 1,008 on-site respondents participated. The inclusion criteria were to be of legal age and have a good understanding of Spanish. To balance the sample in age and sex, three of the researchers were organized in a district sampling. The nature of the study was explained to them, and they were asked for informed consent to participate and agreed to be followed up later (T1 and T2) by selecting the method (WhatsApp or Email). The questionnaire was collected using a self-administered electronic format. This research was approved by the Ethics Committee of the Universidad Rey Juan Carlos (Registration number: 0103202006520).

At T0, demographic data (age, sex and educational level) and the scale of perceived vulnerability to disease were collected (Online Appendix).

At T1, from May 4–11, 2020, the total lockdown had been completed in Spain and dental clinics, which had remained open only for dental treatment during the lockdown, were allowed to reopen. All T0 participants were contacted at T1, all T1 participants were contacted at T2. The sample loss at T1 was 4.6% (961), participants no wished to participate. Through Google forms, participants filled out an informed consent for participation and an online form. To avoid contact, the questionnaire was sent to them by email or WhatsApp. All questions appeared consecutively after accepting participation in the study and entering the participant's identification code.

At T1, the survey consisted of: (1) Perceived vulnerability to disease scale (already collected at T0); (2) COVID-19 fear scale (published after T0, so it was not applied at T0); (3) The question was asked if the dental clinic would be avoided through fear of COVID-19 (Online Appendix).

At T2, Spain had administered at least one dose of the vaccine to workers determined to be essential, risk groups and people over the age of 65 (2–10 May 2021). All T1 participants were contacted to participate in T2. The procedure for the collection of data was the same as T1. There was a 5.6% sample loss due to non-response at T2. Accordingly, the final sample comprised 907 participants.

In this phase, the survey consisted of: (1) Scale of perceived vulnerability to disease (which had already been collected at T0 and T1); (2) Scale of fear of COVID-19 (which had already been collected at T1); (3) They were asked if they were vaccinated and if that vaccination is complete. (4) They were asked if they would avoid the dental clinic through fear of COVID-19; and (5) If they have reduced preventive practice in response to COVID-19. The questionnaire is attached in the Online Appendix.

### Instruments

Perceived vulnerability to disease was assessed using an adaptation to the Spanish language of the 15 items Perceived Vulnerability to Disease (PVD) Scale (18). The PVD uses a 7- point Likert-like response format from 1 (totally disagree) to 7 (completely agree). This scale has the two subscales: of perceived infectability (7 items) and germ aversion (8 items). An example of an item in the “Perceived infectability” subscale is “I am more likely to catch an infectious disease than people in my environment”. An example of an item in the “Germ aversion” subscale is, “I prefer to wash my hands right after shaking someone's hand”. The internal consistency of the PVD scale in the present study was in T0 (α = 0.75), T1 (α = 0.82) and T2 (α = 0.87).

The Spanish version of the fear of COVID-19 scale (FCV-19S) was used to evaluate the participants' fear of COVID-19 (19, 20). This scale comprises seven items. The FCV-19S uses a 5- point Likert-like response format from 1 (strongly disagree) to 5 (strongly agree), where higher scores indicate greatest fear of COVID-19. For instance, “It makes me uncomfortable to think about coronavirus-19”. The internal consistency of the FCV-19S in the present study in T1 and in T2 was α = 0.91 and α = 0.89 respectively.

To register the state of the vaccination they were asked: “Are you vaccinated against COVID-19?” The response format was dichotomous (Yes/No). “Have you had the complete vaccination course?” The response format was dichotomous (Yes/No).

Included among the structured questions about dental clinic avoidance were: “Are you currently avoiding going to the dentist because of the fear of COVID-19?” The response format was dichotomous (Yes/No).

With respect to preventive practice in response to COVID-19 the participants were asked: “Have you relaxed the preventive practice of wearing masks in response to COVID-19?” “Have you relaxed the preventive practice of using disinfectant gel in response to COVID-19?” “Have you relaxed the preventive practice of maintaining social distance in response to COVID-19?” “Have you relaxed the preventive practice of wearing masks with social contacts in response to COVID-19?”

Participants rated the questions using a five-point Likert scale, using the responses 1(not at all) to 5 (extremely). The point scoring of all the questions was added to evaluate the degree of relaxation of the preventive practices with a range from 4 to 20. Higher points indicate a greater cessation of preventive practices.

### Statistical analysis

Statistical analysis used SPSS version 24 (SPSS Inc., Chicago, IL, USA). Data analysis included descriptive statistics and the Kolmogorov–Smirnov test to evaluate the as-sumption of normality, which was confirmed. Paired *T*-tests examined differences in T0–T1–T2 for continuous variables. Pearson's correlation coefficient was used to analyse the association between continuous variables. The difference in the relaxation of preventive measures was evaluated by Student's *t*-test. The chi-square test was used to evaluate the change in dental avoidance between vaccinated and unvaccinated. A 2 × 2 ANOVA was carry out to explore dental visit avoidance and vaccination on fear of COVID-19. Statistical significance was established at *p* < 0.05.

## Results

Table 1 shows that the sample in T0 was composed of 1,008 participants (40% men, 60% women). The mean age of the participants was 38.4 years (± 16.1).

**Table 1:** Sociodemographic characteristics in T0 (*N* = 1008).

	**Total 1008 (100%)**
**Age**	
M (SD)	38.9 (16.6)
**Gender**	
Men *N* (%)	418 (41.50%)
Women *N* (%)	590 (58.50%)
**Education level** *N* (%)	
Primary	125 (12.3%)
Secondary	278 (27.7 %)
Higher education	605 (60 %)

13.3% of the participants (*N* = 126) are vaccinated, however only 4% (*N* = 36) have completed the full course. The group of the vaccinated presents an average age of 62.07 ± 18.35 while the non-vaccinated group presents an average age of 35.38 ± 12.46.

### Perceived infectability, aversion to germs, and fear of COVID-19

Participants reported a significant increase from baseline to T1 in the perceived infectability sub-scale (Cohen's d: 0.72) and in the germ aversion sub-scale (Cohen's d: 0.9). However, there was a significant decrease T1–T2 in the perceived infectability sub-scale (Cohen's d: 0.78), the germ aversion sub-scale was maintained at T1-T2. There was a significant decrease in fear of COVID-19 in T1-T2 (Cohen's d: 0.78). See Table 2.

**Table 2:** Mean, standard deviation, *N* (%) in T0–T1, T1–T2, and significance in T0–T1, T1–T2 for the variables of perceived vulnerability to infection (Infectability subscale and Germ-Aversion Subscale) and fear of COVID-19.

**Variables**	**T0**	**T1**	**T2**	**T0-T1 *p*-value**	**T1-T2**
**Vulnerability to infection**					
Infectability subscale	3.3 (1.1)	4.1 (1.1)	3.2 (1.2)	< 0.001^**^	< 0.001^**^
Germ-Aversion Subscale	3.5 (1.1)	4.5 (1.1)	4.6 (1.2)	< 0.001^**^	0.105
**Fear of COVID-19**		20.8 (6.8)	15.9 (7.2)		< 0.001^**^

** Significance at the 0.01 level.

**Figure 1:**
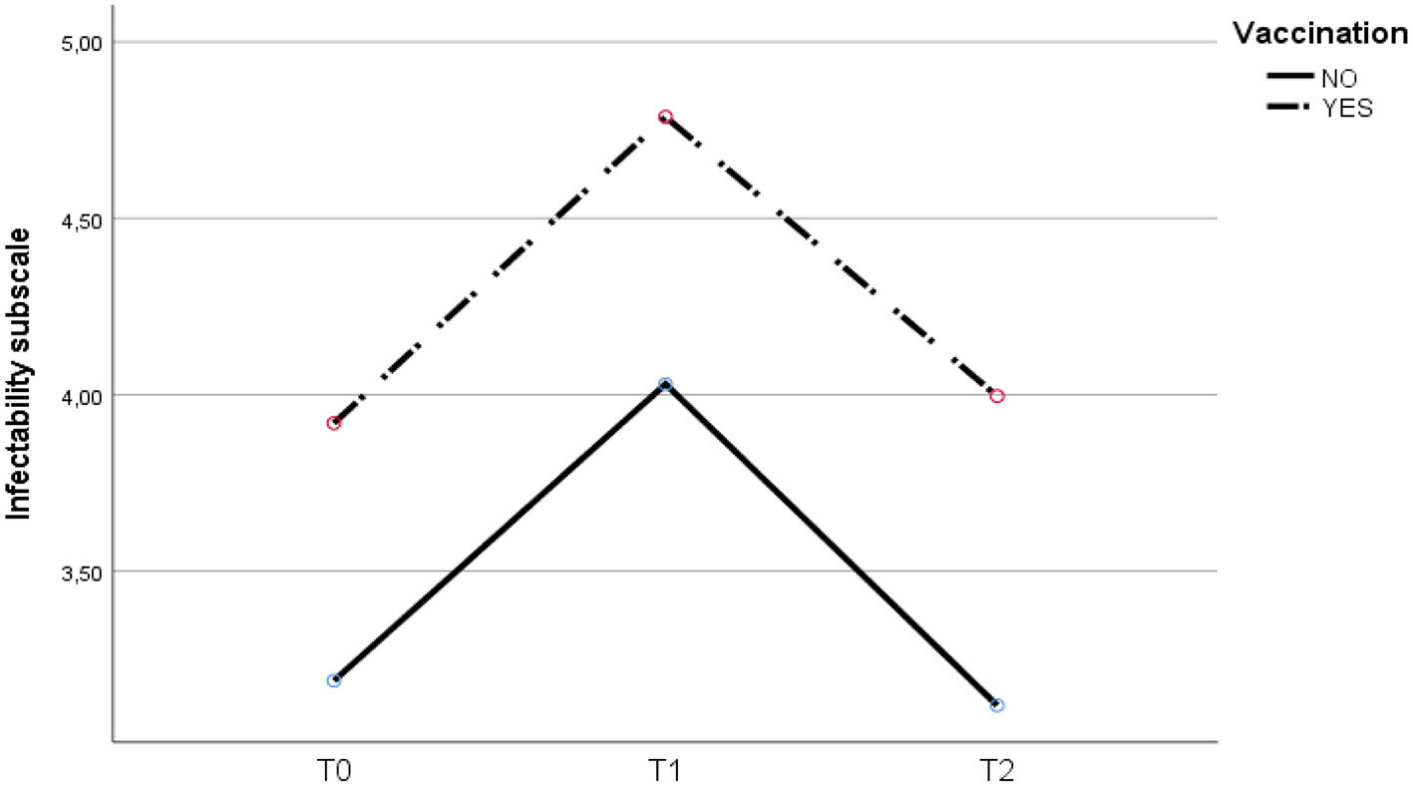
Evolution of perceived infectivity at T0, T1 and T2 for vaccinated and unvaccinated.

**Figure 2:**
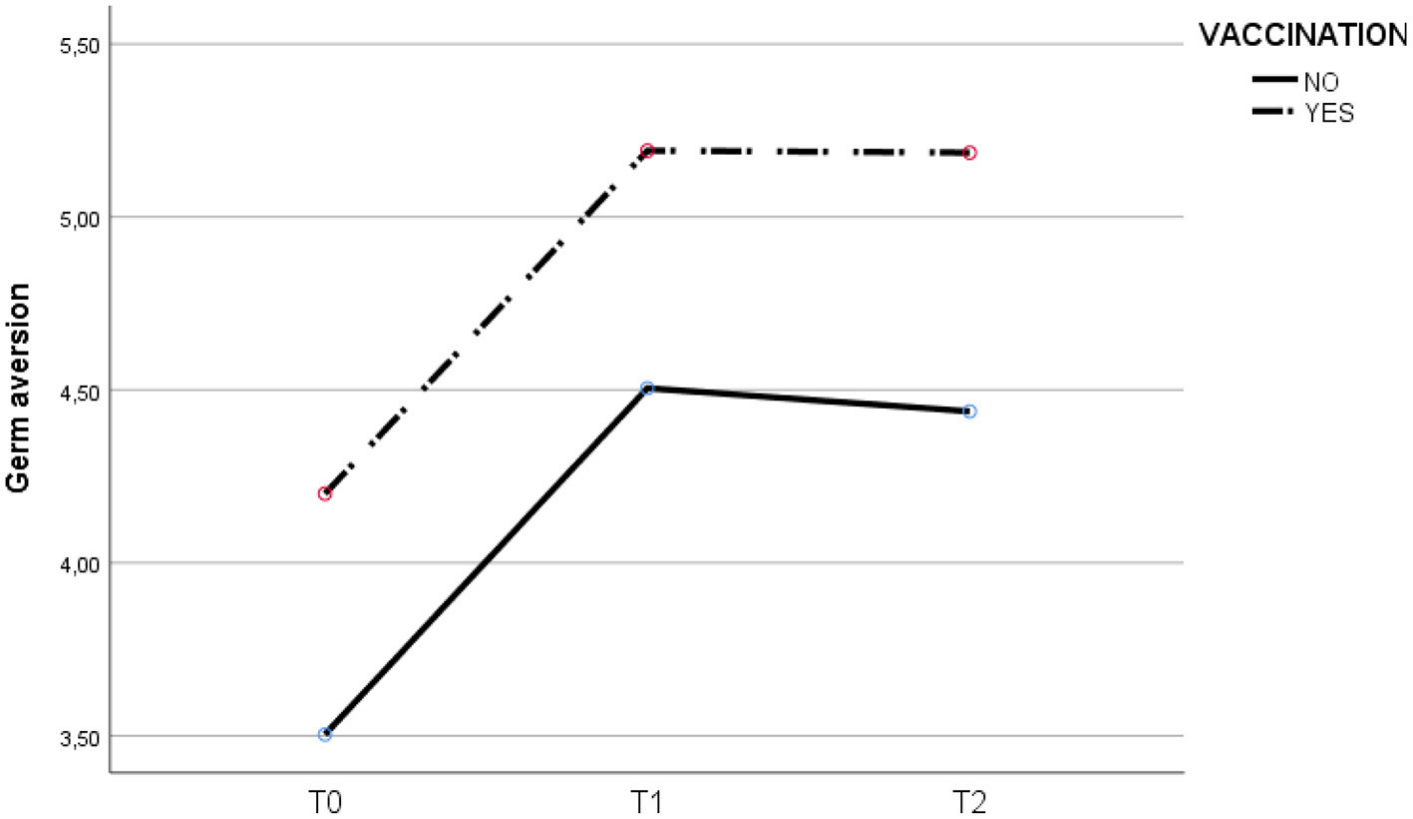
Evolution of germ aversion at T0, T1 and T2 for vaccinated and unvaccinated.

**Figure 3:**
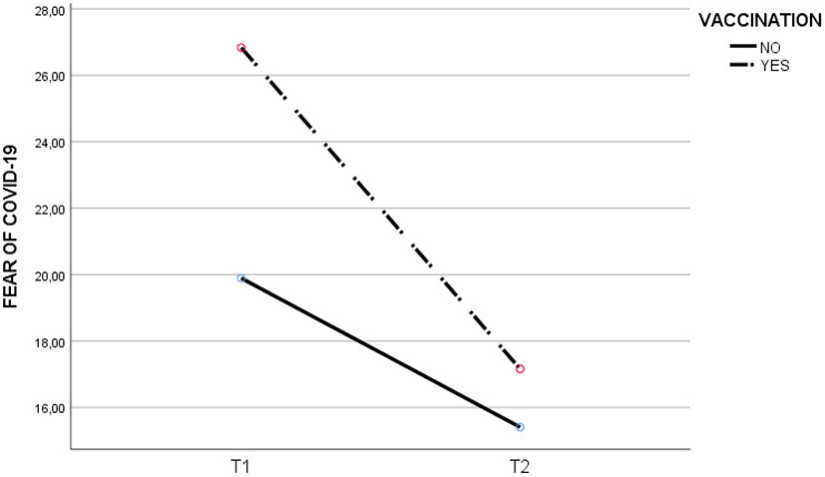
Evolution of COVID-19 fear at T0, T1 and T2 for vaccinated and unvaccinated.

As shown in Table 3, there is a significant positive correlation between the COVID-19 fear scale in T1 and the sub-scales of perceived infectability and germ aversion in T0, T1 and T2 (*p* < 0.01). Furthermore, a strong positive association was found between the germ aversion sub-scale in T2 and perceived infectability in T1 (*r*^2^=0.521, *p* < 0.01) and Germ aversion in T1 (*r*^2^=0.594, *p* < 0.01). Significant differences were found in T1-T2 for the fear of COVID-19. The vaccinated experienced a greater reduction [T1= 26.83 (6.81); T2 = 17.15 (8.35)] than the non-vaccinated [T1= 19.89 (6.29); T2= 15.4 (7.17)]. See Table 4 and Figures 1–3.

**Table 3:** Cronbach's Alpha and intercorrelations between subscale of infectability and germ aversion (T0, T1, T2) and fear of COVID-19 (T1, T2).

**Theoretical range**	**α**	**1**	**2**	**3**	**4**	**5**	**6**	**7**	**8**
1. Infectability subscale T0 (1–7)	0.783		0.284^**^	0.219^**^	0.579^**^	0.229^**^	0.175^**^	0.248^**^	0.047
2. Infectability subscale T1 (1–7)	0.859			0.530^**^	0.190^**^	0.823^**^	0.521^**^	0.323^**^	0.137*
3. Infectability subscale T2 (1–7)	0.765				0.153^**^	0.436^**^	0.352^**^	0.185^**^	0.253^**^
4. Germ aversion subscale T0 (1–7)	0.729					0.208^**^	0.120^**^	0.326^**^	0.120^**^
5. Germ aversion subscale T1 (1–7)	0.771						0.594^**^	0.183^**^	0.113^**^
6. Germ aversion subscale T2 (1–7)	0.763							0.158^**^	0.099^**^
7. Fear of COVID-19 T1 (7–35)	0.913								0.329^**^
8. Fear of COVID-19 T2 (7–35)	0.882								

*Correlation is significant at the 0.05 level,

** Correlation is significant at the 0.01 level.

**Table 4:** Mean, standard deviation and significance according to vaccination for the variables of Vulnerability to infection and Fear of COVID-19 in T0, T1, T2.

**Variables**	**Vaccinated**	**No Vaccinated**	**Vaccinated**	**No Vaccinated**	**Vaccinated**	**No Vaccinated**	**Vaccinated/No Vaccinated**	**Vaccinated/No Vaccinated**
	**M (SD)**	**M (SD)**	**M (SD)**	**M (SD)**	**M (SD)**	**M (SD)**	***p***	***p***
	**T0**	**T0**	**T1**	**T1**	**T2**	**T2**	**T0-T1**	**T1-T2**
**Vulnerability to infection**								
Infectability subscale	3.9 (1.4)	3.1 (1)	4.7 (1.2)	4 (1.1)	3.9 (1.4)	3.1 (1.1)	0.669	0.298
Germ-Aversion Subscale	4.1 (1.4)	3.5 (1)	5.1 (1.2)	4.5 (1.1)	5.1 (1.2)	5.1 (1.2)	0.945	0.557
**Fear of COVID 19**			26.8 (6.8)	19.8 (6.2)	17.1 (8.3)	15.4 (7.1)		< 0.001^**^

** Significance at the 0.01 level.

### Measures of protection against COVID-19

The vaccinated have relaxed their preventive practice (7.78 ± 4.39) in comparison with the non-vaccinated (7.31 ± 1.23) (*t* = 2.461, *p* = 0.014). Nevertheless, no differences exist between those having the complete vaccination (8.75 ± 5.18) or incomplete (8.75 ± 5.18).

### Avoidance of dental visit

As can be seen in Table 5, the non-vaccinated group is a younger group in which dental avoidance in T1 was less than in the vaccinated group [*X*^2^ = 15.33 (*p* < 0.01)]. Nevertheless, the frequency of dental avoidance has diminished in the vaccinated group from T1 to T2 by 68.3% (*N* = 86) while in the non-vaccinated this reduction has only been 4.9% (*N* = 38); *X*^2^ = 18.58 (*p* < 0.01).

**Table 5:** Comparison of the frequency of dental avoidance at T1-T2 in vaccinated (*N* = 126) and unvaccinated (*N* = 781) at T2.

	**T2**			
	**VACCINATED**		**UNVACCINATED**	
T1	I would not go to the dentist *N* (%)	I would go to the dentist *N* (%)	I would not go to the dentist *N* (%)	I would go to the dentist *N* (%)
I would not go to the dentist *N* = 227 (25%)	7 (5.6%%)	86 (68.3%)	96 (12.3%)	38 (4.9%)
I would go to the dentist *N* = 680 (75%)	0 (0%)	33 (26.2%)	28 (3.6%)	619 (79.3 %)
Total *N* = 907 (100%)	7 (5.6%)	119 (94.5%)	124 (15.9%)	657 (84.1%)

An ANOVA 2 × 2 was conducted to explore the avoidance of dental visits and vaccination in response to the fear of COVID-19. The value of relevance for the case of dental visit avoidance is not significant [*F*_(1, 3)_ = 0.072; *p* = 0.39], neither is that for vaccination [*F*_(1, 3)_ = 2.35; *p* = 0.125], however the interaction of vaccination and dental avoidance on the Δ fear of COVID-19 T1-T2 was significant [*F*_(1, 3)_ = 4.92; *p* = 0.027; η^2^ = 0.005].

## Discussion

With this study the evolution of the pandemic has been analyzed from its beginning to the arrival of vaccination as it relates to the fear perception of COVID-19, to the perceived infectability and to germ aversion. The data of this research indicates that a habituation has been produced in the population in response to COVID-19, because, faced with a repeated stimulus the answer is increasingly less intense. This occurs as much with the fear of COVID-19 as it does with perceived infectability (21, 22).

The habituation can be considered the most primitive process of learning and occurs at all levels of the organism, from the cellular to the psychological (23). The aversion to germs has practically remained constant from T1 to T2, however it suffered an important increase from T0 to T1. This can be explained by the lack of knowledge about the modes of transmission of COVID-19 at the beginning of the pandemic, provoking a traumatic situation through the need to maximize hygiene as a protective measure in response to the virus. Also, the excess of information may have been influenced by the mass media. This data agrees with the results found in the bibliography about the increase in germ aversion during lockdown (24). Similar results were also found by Eder et al. (25), showing that aversion to germs is associated with the fear of COVID-19. Due to the lack of efficient treatment, the principal way of reducing the propagation of COVID-19 is preventing the transmission of the virus between people by means of raising awareness, vaccination and the adoption of adequate preventive practices (5, 26, 27). In addition, healthy behaviors that help to improve the prognosis in case of SARS-CoV-2 infection should be promoted, such as: balanced diet, physical activity, avoiding tobacco and alcohol consumption habits (28–31). In Madrid the accumulated incidence remains elevated, 277.19, and the details of this study confirm that preventive practices are being relaxed. This could be explained by two possible causes: the general form of COVID-19 fear has declined, and vaccination has reduced the perception of risk. As well, people balance this less perceived risk by reducing other preventive behavior.

The hypothesis of balancing risk in the context of vaccination conduct was studied in Lyme's disease although no complete inhibition free behavior was found (32). However, the reduction of perceived risk may not be the prime driver of risk behavior. There is also the perception of benefits, a belief that restrictive behavior is associated with contagion and illness and a belief in the efficiency of the vaccine and closer social contact play very important roles.

The limitations of this study are linked to the sample used. It is a sample of convenience and not representative and therefore its results cannot be extrapolated. A possible second limitation comes from the using of measures of auto-information whose answers are based more on social desirability than reality. The third limitation is associated with methodology and the implementation of non-standardized measures to register the preventive behavior of the participants and not permit, through design limitations, the establishment of causal relationships in all of the results. Because of this future line of research will be required to confirm the results. However, the similarities of the results of this study lead us to think that the findings provided can contribute to the current pandemic debate. It should not be forgotten that the population is found on a world stage upon which viral variants are increasing. Several of these are being studied for their greater potential for contagion and severity and for the probability that they can elude the protection that currently approved vaccines have conferred.

For this reason, this study endorses the need for undertaking adequate interventions directed toward the promotion of health, to raising awareness of the measures of preventive practice, healthy behaviors and to the acceleration of vaccination.

## Conclusion

In summary, vaccination has had an impact in the reduction of perceived infectability and in reducing fear of COVID-19. Nevertheless, germ aversion has remained stable and independent of vaccination. Empirical support is also found for the affirmation that vaccination can reduce certain preventive behavior and dental avoidance.

## Data availability statement

The raw data supporting the conclusions of this article will be made available by the authors, without undue reservation.

## Ethics statement

The study was conducted according to the guidelines of the Declaration of Helsinki and approved by the Ethics and Research Committee of REY JUAN CARLOS UNIVERSITY (Registration number: 0103202006520 and 03/03/2020). The patients/participants provided their written informed consent to participate in this study.

## Author contributions

MC-D and MG-O: Conceptualization, formal analysis, data curation, and writing—original draft preparation. MC-D, MG-O, MR-M, and BD-R: investigation. MR-M and BD-R: resources. MR-M: supervision. RG and MR-M: writing—review and editing. RG: methodology. All authors have read and agreed to the published version of the manuscript.

## Conflict of interest

The authors declare that the research was conducted in the absence of any commercial or financial relationships that could be construed as a potential conflict of interest.

## Publisher's note

All claims expressed in this article are solely those of the authors and do not necessarily represent those of their affiliated organizations, or those of the publisher, the editors and the reviewers. Any product that may be evaluated in this article, or claim that may be made by its manufacturer, is not guaranteed or endorsed by the publisher.
