# Supplementary material for: The impact of vaccination upon dental clinic avoidance and the cessation of individual protection measures
Source: Front Public Health. 2022 Sep 21;10:864783. doi: 10.3389/fpubh.2022.864783 (PMC9533680; doi:10.3389/fpubh.2022.864783)
Supplement: Supplementary file 1 [file Data_Sheet_1.docx]

T0 Questionnaire

*Mandatory

1. Insert a code (please, enter your last two digits of your identity card, then the initial of your first name followed by the initial of your last name) *
2. Age *
3. Gender *

Male

Female

1. Level of education *

Uneducated

Primary

Secondary

University degree

**Are you agree with the following statements?:**

1. It really bothers me when people sneeze without covering their mouths *

1 2 3 4 5 6 7

Totally disagree Completely agree

1. If an illness is ‘going around’, I will get it *

1 2 3 4 5 6 7

Totally disagree Completely agree

1. I am comfortable sharing a water bottle with a friend *

1 2 3 4 5 6 7

Totally disagree Completely agree

1. I do not like to write with a pencil someone else has obviously chewed on *

1 2 3 4 5 6 7

Totally disagree Completely agree

1. My past experiences make me believe I am not likely to get sick even when my friends are sick *

1 2 3 4 5 6 7

Totally disagree Completely agree

1. I have a history of susceptibility to infectious disease *

1 2 3 4 5 6 7

Totally disagree Completely agree

1. I prefer to wash my hands pretty soon after shaking someone’s hand *

1 2 3 4 5 6 7

Totally disagree Completely agree

1. In general, I am very susceptible to colds, flu and other infectious diseases *

1 2 3 4 5 6 7

Totally disagree Completely agree

1. I dislike wearing used clothes because you do not know what the last person who wore it was like *

1 2 3 4 5 6 7

Totally disagree Completely agree

1. I am more likely than the people around me to catch an infectious disease *

1 2 3 4 5 6 7

Totally disagree Completely agree

1. My hands do not feel dirty after touching money *

1 2 3 4 5 6 7

Totally disagree Completely agree

1. I am unlikely to catch a cold, flu or other illness, even if it is ‘going around *

1 2 3 4 5 6 7

Totally disagree Completely agree

1. It does not make me anxious to be around sick people *

1 2 3 4 5 6 7

Totally disagree Completely agree

1. My immune system protects me from most illnesses that other people get *

1 2 3 4 5 6 7

Totally disagree Completely agree

1. I avoid using public telephones because of the risk that I may catch something from the previous user *

1 2 3 4 5 6 7

Totally disagree Completely agree

T1 Questionnaire

*Mandatory

1. Insert a code (please, enter your last two digits of your identity card, then the initial of your first name followed by the initial of your last name) *

**Are you agree with the following statements?:**

1. It really bothers me when people sneeze without covering their mouths *

1 2 3 4 5 6 7

Totally disagree Completely agree

1. If an illness is ‘going around’, I will get it *

1 2 3 4 5 6 7

Totally disagree Completely agree

1. I am comfortable sharing a water bottle with a friend *

1 2 3 4 5 6 7

Totally disagree Completely agree

1. I do not like to write with a pencil someone else has obviously chewed on *

1 2 3 4 5 6 7

Totally disagree Completely agree

1. My past experiences make me believe I am not likely to get sick even when my friends are sick *

1 2 3 4 5 6 7

Totally disagree Completely agree

1. I have a history of susceptibility to infectious disease *

1 2 3 4 5 6 7

Totally disagree Completely agree

1. I prefer to wash my hands pretty soon after shaking someone’s hand *

*.*

1 2 3 4 5 6 7

Totally disagree Completely agree

1. In general, I am very susceptible to colds, flu and other infectious diseases *

1 2 3 4 5 6 7

Totally disagree Completely agree

1. I dislike wearing used clothes because you do not know what the last person who wore it was like *

1 2 3 4 5 6 7

Totally disagree Completely agree

12. I am more likely than the people around me to catch an infectious disease *

1 2 3 4 5 6 7

Totally disagree Completely agree

1. My hands do not feel dirty after touching money *

1 2 3 4 5 6 7

Totally disagree Completely agree

1. I am unlikely to catch a cold, flu or other illness, even if it is ‘going around *

1 2 3 4 5 6 7

Totally disagree Completely agree

1. It does not make me anxious to be around sick people *

1 2 3 4 5 6 7

Totally disagree Completely agree

1. My immune system protects me from most illnesses that other people get *

1 2 3 4 5 6 7

Totally disagree Completely agree

1. I avoid using public telephones because of the risk that I may catch something from the previous user *

1 2 3 4 5 6 7

Totally disagree Completely agree

**Please answer the following questions honestly:**

1. I am most afraid of coronavirus-19 *

1 2 3 4 5

Strongly disagree Strongly agree

1. It makes me uncomfortable to think about coronavirus-19 *

1 2 3 4 5

Strongly disagree Strongly agree

1. My hands become clammy when I think about coronavirus-19 *

*.*

1 2 3 4 5

Strongly disagree Strongly agree

1. I am afraid of losing my life because of coronavirus-19 *

1 2 3 4 5

Strongly disagree Strongly agree

1. When watching news and stories about coronavirus-19 on social media, I become nervous or anxious *

1 2 3 4 5

Strongly disagree Strongly agree

1. I cannot sleep because I’m worrying about getting coronavirus-19 *

1 2 3 4 5

Strongly disagree Strongly agree

1. My heart races or palpitates when I think about getting coronavirus-19 *

1 2 3 4 5

Strongly disagree Strongly agree

1. Are you currently avoiding going to the dentist because of the fear of COVID-19? *

Yes No

T2 Questionnaire

*Mandatory

1. Insert a code (please, enter your last two digits of your identity card, then the initial of your first name followed by the initial of your last name) *
2. Have you relaxed the preventive practice of wearing masks in response to COVID-19? *

1 2 3 4 5

Not at all Extremely

1. Have you relaxed the preventive practice of using disinfectant gel in response to COVID-19?*

1 2 3 4 5

Not at all Extremely

1. Have you relaxed the preventive practice of maintaining social distance in response to COVID-19? *

1 2 3 4 5

Not at all Extremely

1. Have you relaxed the preventive practice of wearing masks with social contacts in response to COVID-19? *

1 2 3 4 5

Not at all Extremely

1. Are you vaccinated against COVID-19?

Yes No

1. Have you had the complete vaccination course?

Yes No

**Are you agree with the following statements?:**

1. It really bothers me when people sneeze without covering their mouths *

1 2 3 4 5 6 7

Totally disagree Completely agree

1. If an illness is ‘going around’, I will get it *

1 2 3 4 5 6 7

Totally disagree Completely agree

1. I am comfortable sharing a water bottle with a friend *

1 2 3 4 5 6 7

Totally disagree Completely agree

1. I do not like to write with a pencil someone else has obviously chewed on *

1 2 3 4 5 6 7

Totally disagree Completely agree

1. My past experiences make me believe I am not likely to get sick even when my friends are sick *

1 2 3 4 5 6 7

Totally disagree Completely agree

1. I have a history of susceptibility to infectious disease *

1 2 3 4 5 6 7

Totally disagree Completely agree

1. I prefer to wash my hands pretty soon after shaking someone’s hand *

*.*

1 2 3 4 5 6 7

Totally disagree Completely agree

1. In general, I am very susceptible to colds, flu and other infectious diseases *

1 2 3 4 5 6 7

Totally disagree Completely agree

1. I dislike wearing used clothes because you do not know what the last person who wore it was like *

1 2 3 4 5 6 7

Totally disagree Completely agree

12. I am more likely than the people around me to catch an infectious disease *

1 2 3 4 5 6 7

Totally disagree Completely agree

1. My hands do not feel dirty after touching money *

1 2 3 4 5 6 7

Totally disagree Completely agree

1. I am unlikely to catch a cold, flu or other illness, even if it is ‘going around *

1 2 3 4 5 6 7

Totally disagree Completely agree

1. It does not make me anxious to be around sick people *

1 2 3 4 5 6 7

Totally disagree Completely agree

1. My immune system protects me from most illnesses that other people get *

1 2 3 4 5 6 7

Totally disagree Completely agree

1. I avoid using public telephones because of the risk that I may catch something from the previous user *

1 2 3 4 5 6 7

Totally disagree Completely agree

**Please answer the following questions honestly:**

1. I am most afraid of coronavirus-19 *

1 2 3 4 5

Strongly disagree Strongly agree

1. It makes me uncomfortable to think about coronavirus-19 *

1 2 3 4 5

Strongly disagree Strongly agree

1. My hands become clammy when I think about coronavirus-19 *

*.*

1 2 3 4 5

Strongly disagree Strongly agree

1. I am afraid of losing my life because of coronavirus-19 *

1 2 3 4 5

Strongly disagree Strongly agree

1. When watching news and stories about coronavirus-19 on social media, I become nervous or anxious *

1 2 3 4 5

Strongly disagree Strongly agree

1. I cannot sleep because I’m worrying about getting coronavirus-19 *

1 2 3 4 5

Strongly disagree Strongly agree

1. My heart races or palpitates when I think about getting coronavirus-19 *

1 2 3 4 5

Strongly disagree Strongly agree

1. Are you currently avoiding going to the dentist because of the fear of COVID-19? *

Yes No
